# Immunomodulation for optimal cardiac regeneration: insights from comparative analyses

**DOI:** 10.1038/s41536-021-00118-2

**Published:** 2021-02-15

**Authors:** Luiza Farache Trajano, Nicola Smart

**Affiliations:** British Heart Foundation Centre of Regenerative Medicine, Burdon Sanderson Cardiac Science centre, Department of Physiology, Anatomy and Genetics, University of Oxford, Oxford, UK

**Keywords:** Regeneration, Cell death and immune response

## Abstract

Despite decades of research, regeneration of the infarcted human heart remains an unmet ambition. A significant obstacle facing experimental regenerative therapies is the hostile immune response which arises following a myocardial infarction (MI). Upon cardiac damage, sterile inflammation commences via the release of pro-inflammatory meditators, leading to the migration of neutrophils, eosinophils and monocytes, as well as the activation of local vascular cells and fibroblasts. This response is amplified by components of the adaptive immune system. Moreover, the physical trauma of the infarction and immune-mediated tissue injury provides a supply of autoantigens, perpetuating a cycle of autoreactivity, which further contributes to adverse remodelling. A gradual shift towards an immune-resolving environment follows, culminating in the formation of a collagenous scar, which compromises cardiac function, ultimately driving the development of heart failure. Comparing the human heart with those of animal models that are capable of cardiac regeneration reveals key differences in the innate and adaptive immune responses to MI. By modulating key immune components to better resemble those of regenerative species, a cardiac environment may be established which would, either independently or via the synergistic application of emerging regenerative therapies, improve functional recovery post-MI.

## Introduction

Myocardial infarction (MI) causes the death of ~1 billion cardiomyocytes (CM)^1^. As the human heart is incapable of regeneration, CM are replaced by a non-contractile scar, leading to structural changes and functional decline, culminating in heart failure (HF)^2^. Patients suffering from HF face debilitating morbidity and a 5-year mortality rate of 50%^3^. Given that cardiovascular diseases are the number one cause of mortality worldwide^4^, costing over US$900 billion per annum^5^, there is an urgent need to tackle the clinical and economic burden of HF. Current management of HF relies on the use of *β*-blockers, diuretics and vasodilators, as well as surgical procedures to implant assist devices^6^. These interventions do not prevent disease progression or restore function, leaving a need for curative options and an ultimate goal to fully regenerate the human heart.

Achieving regeneration will require stimulating CM replenishment and neovascularisation, as well as ensuring electromechanical stability and resolving fibrosis. While immune cell infiltration is needed to clear necrotic cells, initiate angiogenesis and promote fibroblast growth, a rapid resolution and avoidance of autoreactivity is essential for preventing excessive damage^7^. Over the past two decades, researchers have identified several potential strategies to trigger regeneration. A discussion of these approaches is beyond the scope of this article, however the advances and current challenges in this field have been reviewed at length elsewhere^8–10^. Despite promising results in pre-clinical models, clinical trials have been either disappointing or inconsistent and none are yet clinically applicable^11^. Experimental therapies face obstacles including delivery, efficacy, rejection, as well as the common hurdle of a hostile immune response^12–15^.

This review will provide an overview of experimental animal models that have informed our understanding of cardiac repair mechanisms, before focusing on the innate and adaptive immune response to MI. By comparing the immune response in humans to those of model organisms that are capable of undergoing substantial cardiac regeneration, key targets for immunomodulation are revealed which may ameliorate the efficacy of currently unsuccessful regenerative approaches.

## Regenerative models

Comparative analyses of model organisms provide insights into the molecular mechanisms of cardiac injury and regeneration (Table 1). Many vertebrate species are capable of achieving a full anatomical and functional recovery of cardiac tissue following injury. These include, for example, the amphibians, the newt and the axolotl^16,17^, as well as the zebrafish^18,19^, which can be compared to its non-regenerating counterpart, the medaka. In addition, the Mexican cavefish *Astyanax mexicanus* produced two populations following independent evolution: those which are cave-dwelling and those which live on the surface, only the latter of which are capable of regenerating following ventricular resection^20^, thus allowing for an analysis which circumvents interspecies difference. Mammalian models display only transient regenerative capabilities, which decrease shortly after birth. In a seminal paper, Porrello et al. showed that the heart of postnatal day (P) 1 mouse can regenerate with minimal hypertrophy and fibrosis following partial surgical resection, however, this capacity is lost by P7^21^. This is mirrored in porcine hearts, which show a sustained recovery of cardiac function following permanent coronary artery ligation at P2, however, regenerative potential was similarly lost, with predominantly fibrotic repair at P14^22^ and P30^23^.

**Table 1:** The major animal models used to study cardiac regeneration.

Model	Regenerative capacity across lifespan	Injury model used	Additional information
**Zebrafish**	Full regenerative capacity	- Apical resection - Cryoinjury - Genetic ablation	- The zebrafish can be compared to the medaka, another teleost fish which is incapable of cardiac regeneration
**Amphibians** Newt and Axolotl	Full regenerative capacity	- Basal/apical resection	- Capable of regenerating many body parts (e.g., limbs)
**Mexican cavefish**	Cave-dwelling: No regeneration Surface-dwelling: Full regenerative capacity	- Apical resection	- Allows for same-species comparison of regenerative and non-regenerative cohorts
**Mouse**	Regenerative capacity decreases after birth	- Apical resection - Cryoinjury - Genetic ablation - Coronary ligation	- Postnatal day (P) 1 mouse is capable of cardiac regeneration - This capacity is lost by P7 - Mammalian model, therefore more relevant to humans
**Pig**	Regenerative capacity decreases after birth	- Coronary ligation	- Recovery of cardiac function post-injury at P2 but not P30 - Mammalian model and therefore more relevant to humans

The zebrafish, amphibians, and surface-dwelling Mexican cavefish retain their capacity for cardiac regeneration throughout life. Within the first weeks of life, the mouse and pig lose the ability to achieve a complete cardiac regeneration. The cave-dwelling adult Mexican cavefish is unable to regenerate, irrespective of age.

Importantly, studying animal models of regeneration has demonstrated that the nature of the immune response is critical in achieving regeneration. Glucocorticoid-mediated suppression of the zebrafish immune response is associated with a lack of regeneration;^24^ conversely, an enhanced immune response correlates with impaired regeneration in the Pachón cave-dwelling *Astyanax* sub-species^20^. This can be translated to human subjects: Wide-spread immunosuppression by corticosteroids, methotrexate, intravenous immunoglobulin G and cyclosporin A have led to widely inconsistent results in clinical trials. Importantly, some studies suggest that these interventions have a negative impact on the clearance of necrotic cells and a disruption of post-infarct healing^25^. Thus, harnessing the regenerative properties of the immune system is not as simple as upregulating or downregulating the entire response: the post-MI immune response must be carefully fine-tuned.

## The innate immune response to MI

The inflammatory response to MI, referred to as sterile inflammation, proceeds through three distinct but overlapping phases: inflammation, proliferation, and resolution.

The timeframe for these events varies according to the type of injury and model species. For the adult mouse ischaemia-reperfusion model^26^, which most closely reproduces human MI, a marked induction of cytokines, signalling the start of the inflammatory phase, occurs within 6 h of reperfusion and lasts for 3 days. Following the seventh day of reperfusion, the proliferative phase commences, marked by an increase in macrophage and myofibroblast density. After 28 days of reperfusion, a scar is formed, denoting resolution of the injured region^26^.

## Inflammatory phase

### Triggering the inflammatory response

**Fig. 1:**
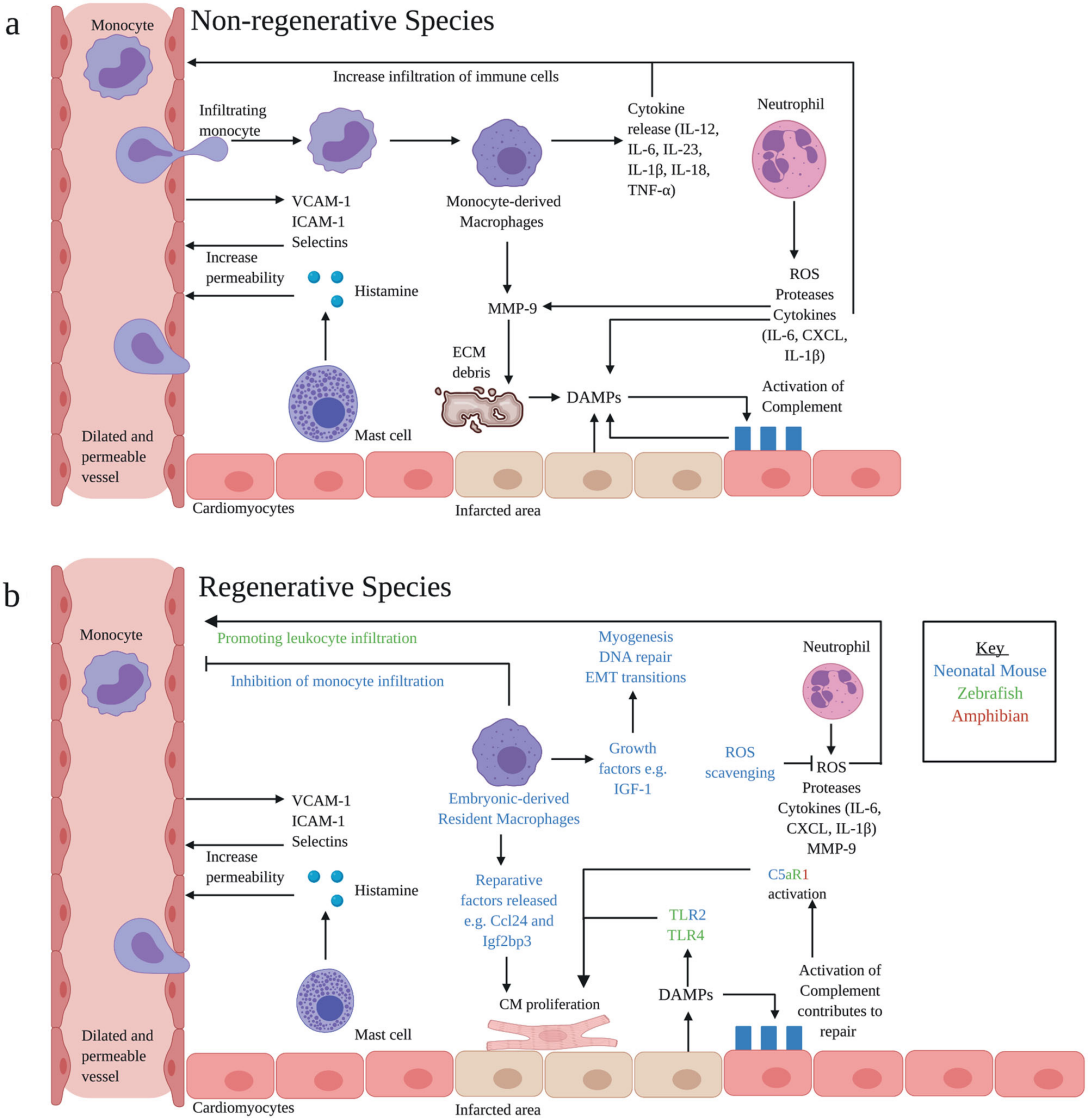
Inflammatory phase: key processes and pathways. **a** DAMPs arise from the infarcted tissue. DAMPs trigger TLR and NLR expressed on CM and resident immune cells, resulting in cytokine release. Subsequently, vascular endothelial cells increase ICAM-1 and V-CAM-1 expression and mast cells increase histamine production. This leads to an increase in vascular permeability, allowing leucocytes to infiltrate. Neutrophils release ROS, proteases, cytokines and MMP-9. Infiltrating M1macrophages release cytokines and MMP-9. Factors released by the macrophages and neutrophils cause damage to the ECM, exacerbating injury and thereby producing more DAMPs. DAMPs are able to activate complement. Complement clears damaged cells; however, over-stimulation of the complement system produces more DAMPs and augments inflammation. **b** There are clear differences in animal models capable of regeneration: (i) complement contributes to CM proliferation and reparative functions, C5aR1 is of particular importance (ii) CCR2^−^ Embryonic-derived macrophages are present following injury, contributing to inflammation resolution and regeneration (iii) ROS scavenging prolongs the proliferative window in neonatal mice. ROS promotes leucocyte infiltration in the zebrafish. DAMPs, damage-associated molecular patterns; TLR, Toll-like receptors; NLR, NOD-like receptors; CM, cardiomyocytes; ICAM, intercellular adhesion molecule; VCAM, vascular cell adhesion molecule; MMP, matrix metalloproteinase; ROS, reactive oxygen species; CCR2, C–C chemokine receptor type 2; ECM, extracellular matrix; IL, interleukin; TNFα, tumour necrosis factor α; C5aR1, complement 5a receptor 1. Adapted from ref. ^16^. Created with BioRender.com.

During an MI, restricted blood flow to the myocardium leads to extensive CM death^27^. Histones, nucleic acids, heat-shock proteins and adenosine triphosphate (ATP) from dead cells, as well as fragmented extracellular matrix (ECM) components, act as damage-associated molecular patterns (DAMPs), which trigger Toll-like receptors (TLRs) and NOD-like receptors (NLR)^16,28^. These receptors, expressed on both CM and cardiac-resident immune cells, play a crucial role in activating the inflammasome to evoke IL-1β and IL-18 secretion^29^ (Fig. 1).

The initiation of the immune response is an important part of triggering regeneration in model organisms. In zebrafish, administration of zymosan or lipopolysaccharides (LPS), targeting TLR2 and TLR4, respectively, has been shown to pre-condition CM, augmenting cell-cycle re-entry and cell survival following injury^30^. Moreover, administration of LPS to zebrafish also triggers the expression of the retinoic acid synthesising enzyme Aldh1a2, which is required for CM proliferation during cardiac regeneration^31^. Interestingly, intraperitoneal injection of the TLR3 agonist Poly(I:C) to the non-regenerative medaka promotes macrophage recruitment, revascularisation and CM proliferation^32^. In addition, intramyocardial injection of zymosan A triggers a cardiac regenerative response in neonatal mice;^33^ however, immunosuppression inhibits this response. Taken together, this indicates that the initiation of acute inflammation is critical in stimulating regeneration.

### Sensing cardiac damage

Cardiac damage liberates self-DNA, which is sensed by the cyclic GMP-AMP synthase (cGAS)-stimulator of interferon genes (STING) pathway, to further exacerbate the pro-inflammatory response^34^. Although the cGAS-STING pathway is typically upregulated in viral infections, it has been shown, via single cell RNA-sequencing analysis (scRNA-seq) of over 4000 leucocytes from infarcted and non-infarcted murine hearts, that MI also induces this axis. Mice deficient in components of this pathway, either by genetic knockout or depletion with neutralising antibodies, displayed improved survival post-MI, diminished pathological remodelling and preserved organ function, as compared to controls^35^. This was associated with an anti-inflammatory environment and M2 cell polarisation^36^. Transient inhibition of the interferon-dependent response may therefore provide another therapeutic avenue. Anti-interferon therapy has been used in clinical trials for systemic lupus erythematosus^37^, showing improvements in organ-specific disease markers, as well as highlighting the feasibility of targeting this pathway in humans. However, translation into patients post-MI is yet to be explored.

The cGAS-STING pathway remains relatively unexplored in animal models of cardiac regeneration. However, it is of note that cGAS is a dispensable component of this pathway for the zebrafish when detecting viral challenges, such as herpes simplex virus 1^38^. It is possible that the ability to by-pass this component of cytosolic DNA sensing mechanisms is advantageous for regeneration. However, this requires further investigation.

### Complement activation

DAMPs activate the complement system. Complement is critical for the clearance of damaged cells. Knockout of the complement cascade, by targeting complement component 3 (C3) post-injury in adult mice impaired LV function and accelerated development towards HF, associated with increased apoptosis, as well as reduced CM and KIT+ progenitor cell proliferation. Therefore, complement may orchestrate molecular signalling to enhance CM survival, and potentially proliferation, following acute injury, albeit determination of proliferation will require assessment of cytokinesis, in addition to DNA synthesis, and progenitor contribution will necessitate reliable genetic lineage tracing^39,40^. Although activation of complement is considered beneficial, over-stimulation of the pathway could be detrimental, leading to sustained inflammation and damage. The complement lectin pathway plays a key role in chronic HF^41^ and complement neutralisation reduces patient mortality^42,43^.

Importantly, the complement system promotes repair in regenerative models^44^. Zebrafish mount a stronger complement response in comparison to the non-regenerative medaka^44^. The complement receptor, C5aR1, is activated in CM and endothelial cells (EC) following cardiac resection of axolotl, zebrafish and neonatal mice; inhibition of C5aR1 attenuates CM proliferation^45^. Post-MI patients given inhibitors of the C1 receptor alongside thrombolytic therapy showed a decrease in markers of cardiac damage (Troponin T and creatinine kinase-MB). Moreover, patients receiving complement inhibition alongside cardiopulmonary bypass displayed improvements in stroke volume and mean arterial pressure, as well as a reduction in intensive-care and in-hospital stay^25^. Given the indispensability of complement in regenerative models and promising findings in clinical trials, further work should seek to develop interventions which can target this system in a specific, beneficial manner. This may be achieved, for example, by selectively targeting distinct complement pathways.

### Neutrophils exacerbate injury during the inflammatory phase

Coronary EC increase their expression of adhesion molecules (notably ICAM-1 and VCAM-1) following MI, while mast-cell-released histamine increases vascular permeability^46^, collectively increasing leucocyte infiltration. The first cells to be recruited are neutrophils, releasing reactive oxygen species (ROS), proteases and cytokines to augment leucocyte infiltration and proliferation. Excessive neutrophil activity exacerbates injury. For example, neutrophilic secretion of myeloperoxidase (MPO) leads to maladaptive remodelling. MPO deletion in adult mice results in decreased LV dilatation and a significant improvement in LV function, as compared to wild-type (WT) mice^47^. In addition, depletion of endothelial Brahma-related gene (Brg)1, which mediates neutrophil–endothelium adhesion, leads to a decrease in ventricular fibrosis and a better recovery^48^. However, it is important to note that neutrophils are not exclusively deleterious, showing beneficial reparative functions during the proliferative phase, as discussed below.

In model systems, neutrophil activity is an area of particular interest. ROS production in the mouse heart during the first week of birth causes DNA damage and cell cycle arrest in CM. Moreover, ROS scavenging is associated with prolongation of the postnatal proliferative window of CM^49^. This was proposed to account for the decreased regenerative capacity in adult mice in comparison to neonatal mice. Antioxidants, such as N-acetylcysteine (NAC) block ROS post-MI. NAC have been investigated in clinical trials, demonstrating a 5.5% reduction in infarct size compared to placebo, as shown by cardiac magnetic resonance imaging, as well as improvements in LV function. However, it is important to note that clinical trials looking at NAC have been small, and the long-term effects of interventions were not investigated^25^. Conversely, however, hydrogen peroxide, a major ROS, initiates inflammation by rapid leucocyte recruitment in zebrafish, promoting CM proliferation^44^. Therefore, the relationship between redox signalling and regeneration requires further investigation.

### Macrophages are central to regeneration

Monocytes are subsequently recruited, differentiating into macrophages in response to IFN-γ, TNF-α and DAMPs. These macrophages, originally described as the ‘M1’ type, are a highly inflammatory population mobilised from bone marrow during the early stages of myocardial injury. They release pro-inflammatory cytokines and matrix metalloproteinase (MMP)-9, amplifying the response, promoting further cell recruitment and damage to the ECM. It is perhaps unsurprising, therefore, that inhibiting monocyte infiltration is correlated with improved outcomes in murine models^50^.

However, macrophages are essential for cardiac regeneration. Macrophage recruitment is reduced and delayed in the medaka, post-injury, in comparison to the zebrafish. In addition, clodronate liposome depletion of macrophages leads to compromised cardiac regeneration in the zebrafish^32^. Moreover, depletion in P1 mice is associated with an increase in fibrosis^51^. This raises the question as to how the inhibition of macrophage activity can result in both deleterious and beneficial outcomes. A potential explanation is that macrophage populations are heterogeneous and distinct sub-types respond differently post-MI, contributing to contrasting outcomes. Following injury, neonatal mice expand MHC-II^low^CCR2^−^ embryonic-derived resident macrophages, which produce minimal inflammation and inhibit monocyte infiltration^52^. However, following injury in non-regenerating adult mice, monocyte infiltration gives rise to MHC-II^high^CCR2^+^ macrophages, which are pro-inflammatory and lack reparative activity^53^. Diphtheria toxin receptor (DTR)-mediated depletion of MHC-II^low^CCR2^−^ macrophages produced a larger infarct area, exaggerated remodelling and a decrease in systolic function, compared to WT controls. Conversely, depletion of MHC-II^high^CCR2^+^ macrophages results in preserved function^54^.

Further characterisation of signals which regulate monocyte-derived and embryonic-derived macrophage recruitment and activation has potential to provide novel therapeutic insights. For example, transcriptomic and epigenomic analysis revealed that P1 macrophages in neonatal mice preferentially express the secreted cytokine *Ccl24* and *Igf2bp3* RNA-binding protein, in comparison to P14 macrophages. Although the mechanism remains unclear, these molecules are thought to promote regeneration by enhancing postnatal CM proliferation^55^. In addition, scRNA-seq of distinct macrophage subsets reveals that cardiac resident CCR2^−^ cells express several growth factors (i.e., insulin-like growth factor) and genes associated with myogenesis, DNA repair and epithelial-mesenchymal transitions^56^. As a potential therapy, CCR2^−^ macrophage cell infusion post-MI may be beneficial to reinstate regenerative functions in the adult heart.

## Proliferative phase

### Macrophages and fibroblasts participate in scar formation

During the proliferative phase, macrophages transition into a so-called ‘M2’ phenotype, those that ostensibly promote tissue repair. It is crucial to note that the M1/M2 paradigm may be overly simplistic and misleading; as alluded above, considerable heterogeneity is now recognised. Macrophages are likely to constitute a spectrum of mixed phenotypes^57^ and exhibit plasticity, adopting different activity states^58^. For example, scRNA-seq analysis identified a number of distinct macrophage phenotypes associated with regenerative and fibrotic processes^59^.

**Fig. 2:**
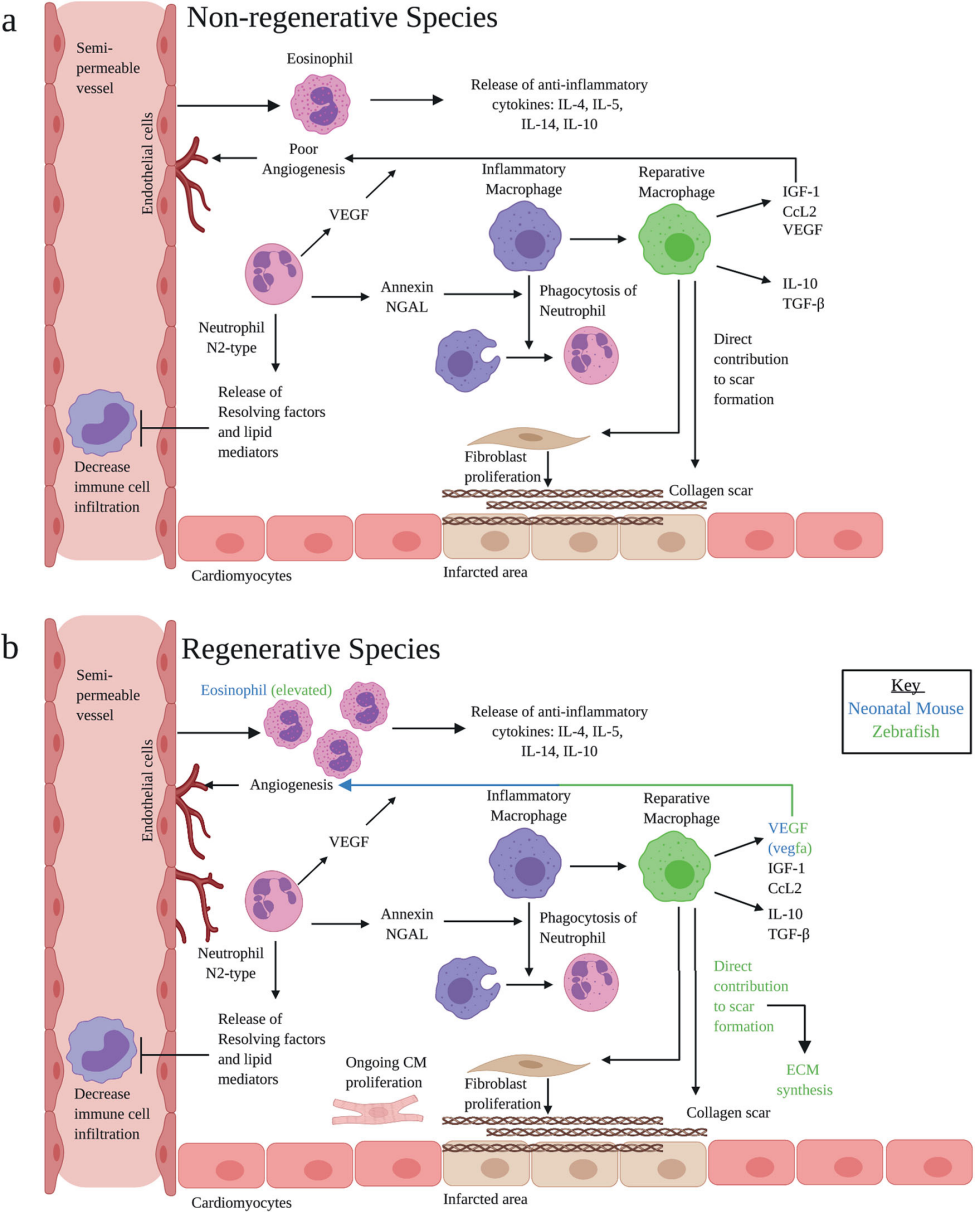
Proliferative phase: key processes and pathways. **a** Inflammatory macrophages phagocytose neutrophils to mark the end of the inflammatory phase. Inflammatory macrophages shift to the reparative phenotype, releasing anti-inflammatory cytokines and VEGF, released by endothelial cells, CM and immune cells, to promote angiogenesis and repair. Reparative macrophages contribute directly to scar formation, as well as triggering fibroblast proliferation. Fibroblasts are responsible for producing the collagen scar. N2-type neutrophils release resolving factors and lipid mediators to decrease immune cell infiltration. Annexin and NGAL, produced by neutrophils, assists in the phagocytosis of pro-inflammatory neutrophils. Eosinophils release anti-inflammatory cytokines. **b** In regenerative species, fibrosis is transient. Eosinophil counts are elevated. M2 cells directly contribute to scar formation, as well as ECM synthesis. VEGF, vascular endothelial growth factor; IL, interleukin; TGF-β, transforming growth factor-β; NGAL, neutrophil gelatinase-associated lipocalin. Adapted from ref. ^16^. Created with BioRender.com.

‘M2’-like, reparative macrophages release anti-inflammatory cytokines, such as IL-10 and TFG-β, as well as VEGF to promote angiogenesis^60^. Moreover, they promote fibroblast activation in order to form a scar^27^ (Fig. 2). Scarring and regeneration are not mutually exclusive events: regenerative models also require the formation of a scar. For example, genetically ablating collagen-1-α-2 expressing cells in zebrafish impairs post-injury CM proliferation^61^. Cardiac regeneration in amphibians is reliant on macrophage-mediated control of fibroblast activation. Depleting macrophages in the context of cardio-cryoinjury in the axolotl limits fibroblast activation, modifies ECM synthesis and blocks regeneration^62^. Remarkably, transcriptomic analysis also reveals collagen upregulation in zebrafish and mouse macrophages post injury and adoptive transfer of macrophages from these species enhances scar formation^63^. Thus, reparative macrophages cells also directly contribute to scar formation, independently of fibroblasts, by releasing collagen. ScRNA-seq identified a new population of cells expressing a hybrid molecular signature of genes from macrophages and fibroblasts^64^, termed fibrocytes^65^. This cell population exhibits high plasticity, adopting the combined phenotype of both cell types to promote repair. Further, detailed characterisation of these key cell types will be pivotal towards advancing our understanding of endogenous repair mechanisms and how they may be therapeutically altered.

### Reparative functions of neutrophils

During the proliferative phase, neutrophils perform beneficial functions, releasing resolving factors, lipid mediators and promoting self-phagocytosis by macrophages. Neutrophil depletion in post-injury mice results in worsened cardiac function, increased fibrosis and an increase in HF biomarkers^66^. Similarly, in clinical trials, treatment with monoclonal antibodies against CD11 or CD18 integrins, attempting to block neutrophil infiltration, have not been encouraging. Three different clinical trials demonstrated no difference in infarct size or flow rate^25^. In addition, neutrophil-depleted mice have altered macrophage-polarisation states. Mechanistically, neutrophil gelatinase-associated lipocalin (NGAL) is known to increase the capacity of cardiac macrophages to engulf apoptotic cells. Treatment with recombinant NGAL was able to restore this macrophage phenotype in neutrophil-deficient mice^67^. Given the diametrically opposing neutrophil response in the inflammatory and proliferative phases, it is postulated that there are distinct subsets of neutrophils at play: N1 being pro-inflammatory, and N2 anti-inflammatory, mirroring the M1/M2 classification of macrophages; albeit this binary classification may similarly oversimplify the heterogeneity of neutrophils^68^. While the N1 type predominate across the post-MI time frame, a burst of N2 neutrophils is produced, peaking at 5–7 days post-MI in the mouse^69^. Neutrophil subsets could also be modulated. Simply blocking neutrophil infiltration to prevent the deleterious effects of the inflammatory phase would preclude the beneficial proliferative phase properties of neutrophils. Therefore, a more nuanced approach to selectively inhibit the migration of N1, expand the N2 sub-type, or both, may be of therapeutic benefit.

### Eosinophils help to establish an anti-inflammatory environment

In WT adult mice, eosinophil recruitment occurs at day 4 post-MI. Mice which are genetically deficient in eosinophils (ΔdblGATA), as well as WT mice with a pharmacological depletion of eosinophils, display greater ventricular dilatation relative to control mice and worse cardiac function seven days post-MI. ΔdblGATA mice have a significant reduction in the anti-inflammatory cytokines L-4, IL-5, IL-13 and IL-10 in comparison to WT mice at day 4 following an MI. The expression of pro-inflammatory mediators, namely IL-18, Ccl5 and TNF-α were elevated in the infarcted region in knockout mice compared to WT at day 7 post-MI. Notably, ΔdblGATA mice had a higher number of pro-inflammatory neutrophils and macrophages. Moreover, the expression of CD206, a marker for macrophage activation was reduced in ΔdblGATA mice but restored by intra-peritoneal eosinophil replenishment^70^. This clearly demonstrates the importance of eosinophils in inflammatory resolution. This is corroborated using patient data: In the clinic, eosinopenia post-MI is associated with exacerbated left ventricular dilatation and adverse events. Patients with a low eosinophil count at day-one following an MI had an increased risk of 6-month all-cause mortality^71^. A lower eosinophil count in patients is associated with more extensive oedema, microvascular obstruction and infarct size, as measured by cardiac magnetic resonance^72^.

The importance of eosinophil recruitment is further emphasised in regenerative models. Eosinophils are rapidly recruited to the injured zebrafish heart. These cells remain elevated from 7-21 days post-injury^73^. In addition, eosinophils are elevated in neonatal mouse hearts post-injury^74^. However, functional studies have yet to assess the impact of these cells on regeneration. Interestingly, treatment of ΔdblGATA mice with IL-4 complexes, which are typically stored within pre-formed eosinophil granules, successfully rescued the adverse cardiac remodelling observed in these mice^70^. IL-4 mediated activation of macrophages is associated with a wound-healing phenotype. Although IL-4 administration was ineffective in WT mice^71^, it may be that patients with a persistently low eosinophil count post-MI would benefit from directed IL-4 treatment.

### Neovascularisation, a critical component of infarct repair, is influenced by the immune response

De novo coronary vessel growth is essential to provide oxygen and nutrients to the highly metabolic, active cells of the healing infarct, and is initiated within hours of myocardial injury. Although the underlying mechanisms are incompletely understood, marked differences have been noted between regenerative models, as well as stark differences with the angiogenesis that occurs in other tissues. For an in-depth discussion, readers may refer to^75^. While angiogenic growth factors, such as vascular endothelial growth factor A (VEGFA), are rapidly induced post-MI, the regulatory pathways downstream of VEGFA were unexpectedly repressed in coronary EC of the infarct border zone of the adult mouse heart^76^. A striking contrast was observed in neonatal MI hearts, in which the VEGFA pathway was selectively active throughout the infarct border zone, but inactive in spared myocardium. Consistent with the requirement for rapid re-vascularisation to uphold regeneration, inhibiting coronary vessel growth by induction of a dominant negative *vegfa* in zebrafish prevented CM proliferation, leading to impaired regeneration and scar retention^77^. Macrophages are critical for cardiac regeneration in neonatal mice. It has been demonstrated that cardiac injury in monocyte/ macrophage-depleted neonatal mice led to defects in neovascularisation and regeneration^51^.

Even in non-regenerating adult mouse hearts, components of the immune system are key in the process of neovascularisation. Early infiltrating inflammatory macrophages initiate the angiogenic process, co-localising with EC tips. These are replaced by reparative macrophages which promote neovascularisation through the release of pro-angiogenic factors such as insulin-like growth factor-1 (IGF-1) and CCL2. Macrophages also contact the anastomosis sites of EC, participating in cell fusion. Activated ECs secrete angiopoetin-2 (Ang2) which binds to Tie2-expressing monocytes/macrophages and increases their angiogenic potential^78^. Neutrophils additionally play an important role in angiogenesis via a multitude of mechanisms. For example, VEGFA released under ischaemic conditions promotes the recruitment of CD49d + VEGFR1^high^ CXCR4^high^ neutrophils with high MMP-9 expression levels, which facilitates rapid angiogenesis at hypoxic regions^78^. However, the role of neutrophils in angiogenesis is ill-defined: neutrophils also secrete high amounts of elastase, inducing EC apoptosis and inhibiting pro-angiogenic effects^79^.

## Resolution phase

**Fig. 3:**
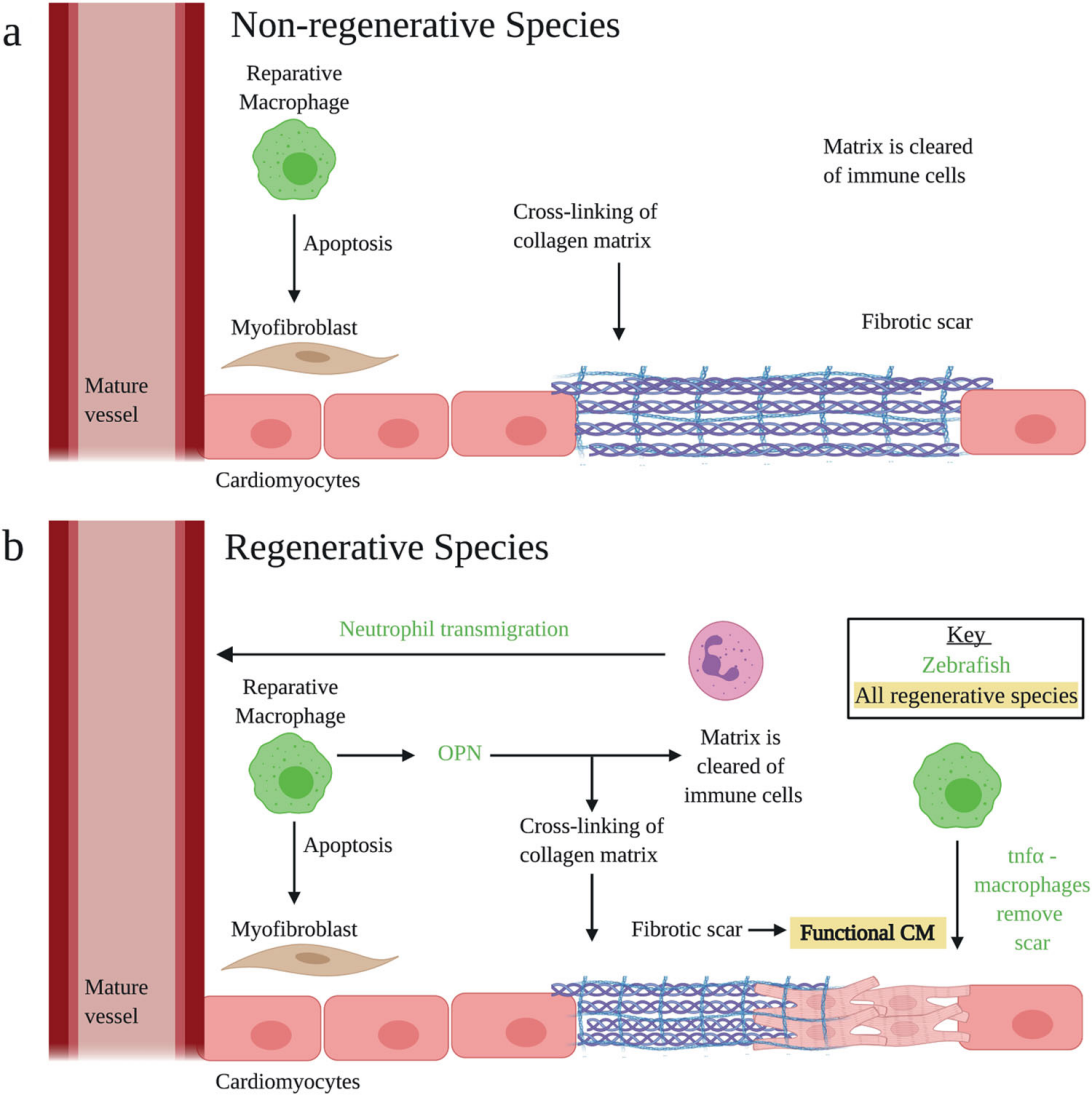
Resolution phase: key processes. **a** The collagen matrix is strengthened, forming a mature fibrotic scar. Immune cells are cleared from the area and M2 cells induce fibroblast apoptosis. In regenerative models, cellular clearance is rapid, and the scar is replaced with contractile tissue. **b** In regenerative models, there are distinctions: (i) Macrophages release OPN which assists in the clearance of immune cells, (ii) Neutrophils transmigrate, (iii) tnf*α–*macrophages remove the scar, (iv) the fibrotic scar is replaced by functional CM. Adapted from ref. ^16^. Created with BioRender.com.

The immature scar is strengthened by the deposition and cross-linking of type-I collagen^80^. Reparative macrophages induce fibroblast apoptosis, and the ECM becomes devoid of cells. The speed of the resolution phase is vital^81^ (Fig. 3): Zebrafish neutrophils rapidly enter the site of injury but are more efficiently cleared in comparison to those in medaka^32^. An important mechanism, observed in the zebrafish using live imaging, is transmigration whereby neutrophils undergo reverse migration from the injured tissue back into the peripheral blood^82^. Prolonged inflammation leads to an increased CM loss, remodelling, fibrosis and rupture. Administration of pro-resolving mediators may shorten the duration of the immune response. For example, Resolvin D1 catalyses resolution in mice to help promote a regenerative environment^83^.

Importantly, scarring is transient in regenerative animal models and the injured area is repopulated by CMs^84,85^. In the zebrafish, tnf*α*+ macrophages promote scar deposition, but a more anti-inflammatory phenotype, tnf*α−*, is necessary to allow scar removal during regeneration. In addition, Osteopontin (OPN), a multifunctional protein, has been shown to be a pro-fibrotic factor downstream of the inflammatory response. OPN is expressed in a subset of macrophages at the site of injury, promoting scarring but also functioning as a regenerative factor by facilitating rapid attenuation of the inflammatory response^73^. Elucidating key cellular phenotypes and proteins associated with scar removal may be of future therapeutic benefit.

## The adaptive immune response to MI

**Fig. 4:**
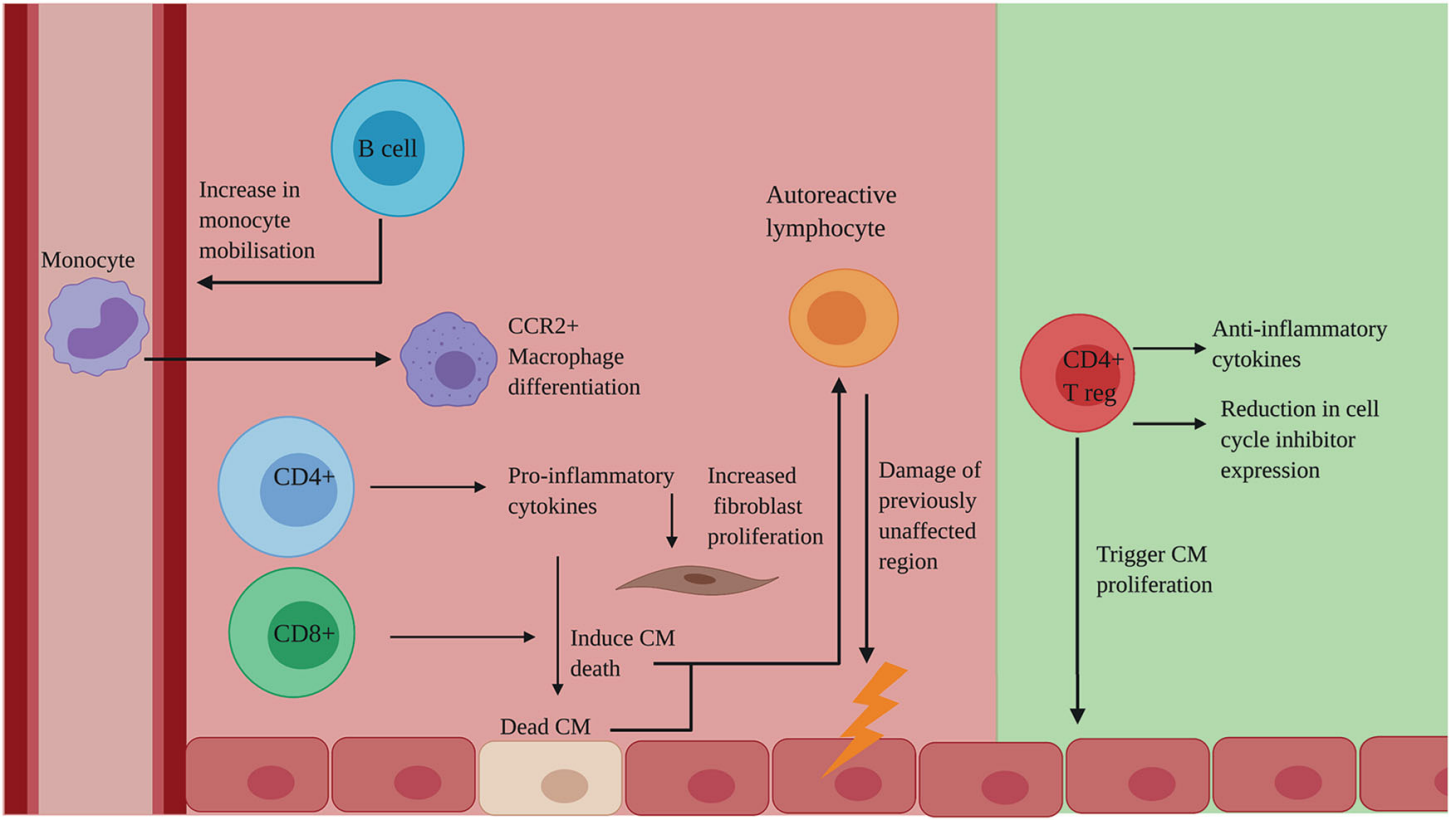
The adaptive immune response to MI: a double-edged sword. Deleterious (red) effects of the adaptive immune system on regeneration: B cells increase monocyte mobilisation, leading to an increase in CCR2+ macrophages. CD4+ T cells produce pro-inflammatory cytokines which induce CM death and increase fibroblast proliferation. CD8+ T cells directly induce CM death. These Dead CM are recognised by autoreactive lymphocytes, leading to damage of previously unaffected regions. Beneficial (green) effects of the adaptive immune system on regeneration: CD4+ Treg cells produce anti-inflammatory cytokines and cause a reduction in cell cycle inhibitors, as well as directly triggering CM proliferation. CCR2, C–C chemokine receptor type 2; CD, cluster of differentiation; CM, cardiomyocytes; Treg, regulatory T cells. Created with BioRender.com.

Although the field has largely focused on the innate response, the adaptive immune system, in which B cells and T cells develop immunological memory to specific antigens, also has an important role to play in post-MI cardiac repair (Fig. 4).

### Lymphocyte involvement in regeneration

B cells infiltrate the mouse myocardium following coronary artery occlusion^86^, producing pro-inflammatory cytokines which contribute to remodelling^87^. This is supported by studies showing that antibody-mediated B cell depletion decreases monocyte mobilisation, resulting in limited injury and improved function^88^. In terms of cell-mediated immunity, CD4^+^ T cells release pro-inflammatory cytokines, which increase CM death and fibroblast proliferation, and CD8^+^ T cells are suggested to have a directly toxic effect on CM^89^.

It has been suggested that the mature and complex adaptive immune system in adult mammals, compared to neonates and other evolutionarily more naïve animals, such as amphibians and fish, may contribute to the limited regenerative capacity. CD4+ T cells in neonatal mice have an ‘intrinsic default mechanism’ to become regulatory T cells (T_reg_) in response to T-cell receptor stimulation. However, this property decreases shortly after birth^44^. T_reg_ have a beneficial role in regeneration, as they release anti-inflammatory cytokines. Moreover, in zebrafish^90^, neonatal mice^91^ and human CM cultures^92^, T_reg_ have been shown to promote CM proliferation. Depletion of T_reg_ in neonatal mice impairs cardiac regeneration, whereas re-infusion of T_reg_ restores regeneration^58^. It is proposed that T_reg_ promote regeneration by reducing the expression of cell-cycle inhibitors^92^, however, this mechanism remains incompletely resolved. The effects of T cells on CM viability can be corroborated by clinical evidence, in that patients with congestive HF have increased levels of pro-inflammatory CD4^+^ T cells (Th1/Th17) and lower frequencies of T_reg_^93^.

### Autoimmunity and tolerance

DAMPs, released from necrotic cells, include cardiac proteins e.g., α-myosin^94^. These proteins may be recognised by autoreactive lymphocytes, triggering an autoimmune response, leading to the destruction of previously unaffected regions of the heart, providing a further supply of autoantigens and thereby catalysing a cycle of autoreactivity^12^. An autoimmune response increases the strain on the remaining CMs to compensate for the loss of others, exacerbating remodelling. In addition, delivery of allogeneic cells into an inflammatory environment may further aggravate immune rejection and impair function of exogenous cells^95^. Current regenerative therapies fail to address the issue of autoreactivity, meaning that tissue destruction is likely to continue, despite intervention^12^.

Dendritic cells (DCs) have recently been identified as a population with potential to modulate the process of post-infarction repair and could be engineered to skew cardiac populations, such as macrophages and T cells, towards tolerance post-MI, a technique utilised in rheumatoid arthritis^96^ and type-1 diabetes^97^. Tolerogenic DCs, treated with TNF-α and cardiac lysate, were injected into post-injury adult mice, incapable of intrinsic regeneration. This led to better wound healing, preserved systolic function and improved survival^98^, associated with a more rapid shift from inflammatory macrophages to reparative macrophages, compared to non-treated mice. However, given that numerous self-antigens, derived from CM, blood vessels or interstitial tissue, may trigger auto-reactivity post-MI, identifying a cocktail of antigens to prime human DC will be key. Although further pre-clinical experiments are required, this proof-of-concept study demonstrates the potential use of tolerogenic DC therapy post-MI.

## Immunomodulation as a regenerative therapy

A landmark study in 2020 revealed that the innate immune response may, in fact, mediate the cardioprotective and pro-regenerative benefits of stem cell therapies. Mice receiving bone marrow mononuclear cells or KIT+ cardiac progenitor cells, the main populations evaluated in clinical trials, displayed enhanced cardiac function post-MI, associated with an upregulation of the acute inflammatory response and enhanced neovascularisation, rather than de novo CM production^99^. This response was characterised by an increase in CCR2^+^ and CX3CR1^+^ macrophages, which altered the activity of cardiac fibroblasts and enhanced the mechanical properties of the injured tissue. The protective effect of this stem cell therapy was abolished when either the immune response or the macrophages were blocked and, importantly, the same response was observed following infusion of dead cells. This demonstrated that the stem cells did not possess any reparative properties per se, but rather, invoked a series of regenerative processes by triggering the innate immune response.

Although these results have only been observed in conjunction with cell-based regenerative therapies, this study raises the question as to whether the field should be focused on altering the immune response to aid efficacy of regenerative therapies or whether immunomodulation is itself sufficient to trigger regeneration. To answer this, further research will be required. As stated, CCR2^+^ macrophages are associated with poor repair. It may be that defining macrophages based on this marker is too narrow in scope and that other phenotypic features are involved in regeneration. Emerging studies, categorising macrophages on the basis of transcriptomic data, as opposed to cell surface markers, may provide the required insights to tailor future therapies. Indeed, profiling adult mouse monocyte/macrophage transitions over the time course of post-MI repair revealed a range of discrete populations with distinct marker profiles, including novel subtypes with apparent specialised roles which include lipid metabolism and iron handling^100^.

## Conclusion

### Limitations and suggested future investigations

It remains unclear as to whether a less hostile immune environment drives regenerative changes in regenerative model organisms or whether the onset of more efficient repair is merely correlated with a decrease in inflammation and autoimmunity. Moreover, as there is limited knowledge regarding precise immune mechanisms during regeneration, one of the challenges for immunomodulation is targeting the correct immune cell population or pathway at the right time^101^. Single-cell ‘-omics’ approaches may reveal specific phenotypes which are associated with regeneration. Targeting specific genes, for example *Ccl24*, associated with embryonic-derived macrophages, will allow interrogation of the specific immune cell sub-populations and pathways important in cardiac regeneration. Nevertheless, targeting may remain a major challenge given that multiple markers are required to define an immune cell type and a deeper understanding of molecular signatures associated with specific cells, as well as technological advances in cell targeting, are required to advance this field. In addition, given the redundant nature of the immune system, it remains unclear to what extent immunomodulation would be beneficial. Blocking one pattern recognition receptor may lead to its role being subverted by another receptor. Testing combination therapies of immunomodulatory drugs in animal models post-MI would be of interest. However, although this may increase efficacy, it may also increase off-target effects.

## Concluding remarks

A hostile inflammatory environment and autoreactivity impedes regeneration. Modulating components of the immune system, to greater resemble those of regenerative model organisms, may ameliorate cardiac repair in human subjects. Although further work is required before translation to the clinic, notable insights into the role of the immune system post-MI have been gleaned through studies of tolerogenic DCs and embryonic-derived macrophages. Harnessing the potential of the immune system may grant us the ability to overcome a roadblock to cardiac regeneration and, thus, unlock the secret to staying young at heart.
